# Alteration of cardiac structure and function and its prognostic value in patients with Takayasu arteritis: a cardiac magnetic resonance study

**DOI:** 10.3389/fcvm.2024.1475535

**Published:** 2024-09-19

**Authors:** Zehui Tang, Chuangwei Wei, Wenjing Zhao, Dongting Liu, Jiayi Liu, Huai Qin, Lili Pan, Nan Zhang, Zhaoying Wen

**Affiliations:** ^1^ Department of Medical Imaging, Beijing Anzhen Hospital, Capital Medical University, Beijing, China; ^2^ Department of Ultrasound, Beijing Anzhen Hospital, Capital Medical University, Beijing, China; ^3^ Department of Rheumatology and Immunology, Beijing Anzhen Hospital, Capital Medical University, Beijing, China

**Keywords:** Takayasu arteritis, cardiac magnetic resonance, late gadolinium enhancement, characteristic, prognosis

## Abstract

**Purpose:**

To investigate the prevalence and characteristics of late gadolinium enhancement (LGE) by cardiac magnetic resonance (CMR) and its prognostic value in patients with Takayasu arteritis (TA).

**Materials and methods:**

Sixty TA patients with a CMR examination were retrospectively included. All TA patients were divided into with LGE-positive and LGE-negative groups. Bi-ventricular function and location, distribution, and pattern of left ventricular (LV) LGE were evaluated in both LGE-positive and LGE-negative groups. Primary outcome was defined as a composite of cardiovascular death, hospitalization for heart failure, coronary artery revascularization, and stroke. Univariate and multivariate Cox proportional hazard regression analyses were used to evaluate the association between variables and primary outcomes.

**Results:**

Sixty consecutive TA patients were enrolled in this study. The mean age was 38.2 ± 13.8 years and 54 patients (54/60, 90.0%) were female. LGE-positive was observed in twenty-one (21/60, 35%) patients in the total patients with TA. LGE was predominantly distributed in the middle wall and subendocardial. The patchy and infarcted LGE patterns were the most common. Compared with the LGE-negative group, the LGE-positive group had reduced LV ejection fraction (*P* = 0.033), elevated LV end-diastolic volume index (*P* = 0.008), LV end-systolic volume index (*P* = 0.012), and LV mass (*P* = 0.008). During a median follow-up period of 1,892 days (interquartile range: 1,764–1,988 days), the primary outcomes occurred in thirteen patients. In the univariate analysis, LGE-positive (hazard ratio [HR] = 4.478, 95% confidence interval [CI]: 1.376–14.570; *P* = 0.013) were independently associated with the primary outcomes. However, LGE-positive did not retain its value as an independent predictor of primary outcomes in the multivariate analysis. Instead, LVMI (HR = 1.030, 95%CI: 1.013–1.048; *P* = 0.001) was the strongest independent predictor of primary outcomes in patients with TA. The Kaplan-Meier plot revealed that patients with LVMI ≥ 57.5 g/m^2^ have a worse prognosis.

**Conclusion:**

LGE-positive detected by CMR was observed in 35% of total TA patients with different distributions and patterns. LGE is associated with adverse LV remodeling and worsen cardiac function. However, LVMI rather than LGE can provide independent prognostic information in patients with TA.

## Introduction

Takayasu arteritis (TA) is a rare large-vessel vasculitis of unknown origin primarily involving the aorta, its main branches, and the pulmonary artery (1–3). Inflammatory infiltration of the arteries leads to wall thickening of the involved arteries and subsequent luminal stenosis, occlusion, dilatation, or aneurysm formation (1, 4, 5). The severity of the clinical manifestations of TA is determined by the degree and extent of the affected vascular lesions (5, 6). Involvement of the aorta and pulmonary artery may result in systemic hypertension or pulmonary hypertension (7, 8).

In addition, cardiac abnormalities due to TA are prevalent in patients with TA, including coronary artery, valve, and myocardium (9). During the clinical course of TA, coronary artery involvement results in coronary arteritis, lumen narrowing, and accelerated atherosclerotic process that may lead to myocardial ischemia or infarction. Dilation of the aortic root may lead to aortic regurgitation. Inflammatory infiltration of the myocardium may manifest as myocarditis (7, 8, 10). Clinically, cardiac involvement may present as ischemic cardiomyopathy, valvular disease, dilated cardiomyopathy, and congestive heart failure. Cardiac involvement is associated with increased morbidity and mortality and is related to poor prognosis (10, 11). For the clinician, adequate assessment of cardiovascular abnormalities is essential for the management of patients with TA.

Currently, multimodal imaging plays an important role in the clinical evaluation of cardiovascular abnormalities due to TA, including ultrasound, computed tomography angiography, magnetic resonance imaging, and positron emission tomography (12, 13). Cardiac magnetic resonance (CMR) not only depicts the thickening of the aortic wall and the aortic root (14), but more importantly, it provides a comprehensive assessment of cardiac structure, function, and myocardial tissue characteristic. Late gadolinium enhancement (LGE) imaging of CMR after intravenous injection of gadolinium contrast agents achieves a noninvasive assessment of myocardial fibrosis or scar (15–17). In fact, LGE was detected in TA patients by CMR imaging (8). However, The more detailed features of LGE and its potential prognostic value in patients with TA remain unclear.

Thus, This study aimed to (1) describe the prevalence and characteristics of LGE observed on CMR imaging and its correlation with cardiac function parameters in patients with TA, (2) to evaluate whether the LGE observed on CMR is independently associated with adverse cardiovascular events in patients with TA.

## Methods and materials

### Study population

This was a single-center retrospective study cohort. Sixty-three consecutive patients with a clinical diagnosis of TA and who underwent CMR examination from January 2015 to October 2022 were retrospectively enrolled in a single-center institution (Beijing Anzhen Hospital affiliated with Capital Medical University). Diagnosis of TA relied on the diagnostic criteria established by the American College of Rheumatology in 1990 (18). The study was performed in accordance with the Declaration of Helsinki and was approved by the Ethics Committee of Beijing Anzhen Hospital affiliated with Capital Medical University. Written informed consent was obtained from all patients with TA.

Demographic characteristics, hemodynamic indices at admission and clinical history were collected in the electronic medical record system for all TA patients.

### CMR images acquisition

CMR examination was performed on 3 Tesla scanners (MAGNETOM Verio, Siemens Healthcare, Erlangen, Germany; MR750W, General Electric Healthcare, Waukesha, WI, USA; Ingenia CX, Philips, Best, The Netherlands) in a single-center institution (Beijing Anzhen Hospital affiliated with Capital Medical University). All patients were trained in breathing before the CMR examination. ECG gating and dedicated coil are routinely used during CMR examination. CMR scanning protocols comprised conventional cine sequences and LGE sequences. Left ventricular (LV) short-axis from the mitral orifice to the apical range and long-axis (four-chamber, three-chamber, and two-chamber views) cine images were acquired during breath-holding. The parameters for short- and long-axis cine sequences are as follows: field of view (FOV) = 340 × 314 mm, matrix = 256 × 256, repetition time (TR)/echo time (TE) = 4.1/1.35 ms, slice thickness = 8 mm, gap = 0 mm (MAGNETOM Verio); FOV = 300 × 300 mm, matrix = 256 × 256, TE/TR = 3.8/0.5 ms, slice thickness = 8 mm, gap = 0 mm (MR750W); FOV = 270 × 270 mm, matrix = 152 × 119, TE/TR = 2.7/1.37 ms, slice thickness = 8 mm, gap = 0 mm (Ingenia CX).

LGE images covering the left ventricle in short-axis and long-axis views were obtained at 10–15 min after intravenous injection of gadopentetate dimeglumine (Bayer Healthcare) at a dose of 0.1 mmol per kilogram of body weight. The inversion time count sequence was used to find the optimal inversion time (TI) before acquiring the LGE images. The optimal TI was defined as the TI of myocardial signal nulling. Based on the adjusted optimal TI time, LGE images were acquired using a phase-sensitive inversion recovery (PSIR) sequence. The parameters of the PSIR sequence are as below: FOV = 340 × 285 mm, matrix = 256 × 256, TE/TR = 15.60/1.56 ms, slice thickness = 8 mm, gap = 0 mm (MAGNETOM Verio); FOV = 300 × 300 mm, matrix = 256 × 256, TE/TR = 6.20/0.5 ms, slice thickness = 8 mm, gap = 0 mm (MR750W); FOV = 360 × 270 mm, matrix = 256 × 192, TE/TR = 12.44/1.19 ms, slice thickness = 8 mm, gap = 0 mm (Ingenia CX).

### Analysis of CMR functional parameters

LV wall thickness (LVWT) was measured online at the left ventricular end-diastolic mid-segment septal wall on a short-axis view through the Radiology work system. The CMR short-axis cine images were imported offline into the commercial software (CVI42, version 5.14, Circle Cardiovascular Imaging Inc., Calgary, Canada). The end-systolic and end-diastolic phases of both ventricles were detected and identified semi-automatically. The LV endocardial and epicardial outlines were sketched to calculate volume, LV ejection fraction (LVEF), and myocardial mass on a short-axis cine view. Myocardial trabeculae and papillary muscles were counted in volume but excluded from the myocardial mass. Only the right ventricular (RV) endocardial contour was drawn to measure RV volume and EF. However, all volumetric and myocardial mass parameters needed to be normalized by dividing by body surface area.

### Analysis of LGE images

**Figure 1:**
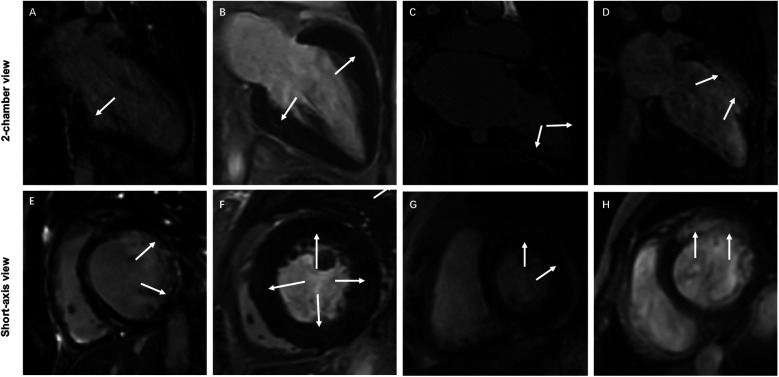
Representative examples of typical characteristics of different LGE distributions and patterns in 4 patients with TA. **(A,E)** Subendocardial infarction LGE (white arrows) in the lateral wall of the basal segment of the left ventricle. **(B,F)** Diffuse patchy LGE (white arrows) in the middle wall at multiple locations in the middle segment of the left ventricle. **(C,G)** Subepicardial LGE (white arrows) at multiple locations in the left ventricular wall. **(D,H)** Transmural infarction LGE (white arrows) in the anterior wall of the basal and middle segments of the left ventricle.

All LGE images were visually evaluated online by two independent double-blind cardiovascular diagnostic radiologists (D.T.L. and J.Y.L.) with more than ten years of experience in the Radiology work system. LGE-positive was defined as hyperintensity within the myocardium was visually observed in any of the sixteen segments of the left ventricle. According to the findings of visual observation, all patients with TA were divided into the LGE-negative and LGE-positive groups. The location, distribution, and pattern of LGE in the sixteen segments of the left ventricle of patients with LGE-positive were visually assessed and recorded. In case of inconsistency, a third cardiovascular diagnostic radiologist (Z.Y.W.) with more than fifteen years of experience visually observed and made a final decision. LGE-positive was categorized into four types according to visually observed distribution of LGE-positive: subendocardial LGE, middle wall LGE, subepicardial LGE, and transmural LGE. LGE-positive was classified into three types based on visually observed patterns of LGE-positive: line LGE, patchy LGE, and infarcted LGE pattern. Representative examples of LGE-positive images are displayed in Figure 1. In addition, the number of LGE-positive segment based on visual observation in each TA patient was also recorded.

### Primary outcome and follow-up

The primary outcome of this study was the occurrence of major adverse cardiovascular events, defined as a composite of cardiovascular death, hospitalization for heart failure, coronary artery revascularization, and stroke. The occurrence of any of the events listed above is defined as reaching the primary outcome. If patients had multiple adverse cardiovascular events, only the first was selected as the primary outcome. The cardiovascular events were obtained by telephone contact or reviewing medical records for overall TA patients. Furthermore, the follow-up time from the date of the CMR examination to the presence of adverse cardiovascular events was calculated.

### Statistical analysis

All continuous data were tested for normality distribution using the Shapiro-Wilk test. Continuous data obeying a normal distribution are expressed as mean ± SD, otherwise as median [interquartile range (IQR)]. Categorical variables are presented as numbers and percentages. The difference between the continuous data of the LGE-negative and LGE-positive groups was compared using the unpaired *t*-test or the Mann–Whitney *U* test (two sides). The difference between the categorical variables of the two groups was analyzed using the Chi-square test or Fisher exact test (two sides). Univariate and multivariate Cox proportional hazards regression analyses were used to assess the effect of various variables on the primary outcome. The association of each variable with outcomes was first evaluated in a univariate analysis by applying a forward stepwise regression approach. Variates with *P* < 0.05 were selected in the univariate analysis and were included in the further multivariate analysis. In order to avoid collinearity between CMR functional parameters (including LVEF, LVEDVI, and LVESVI), the variance inflation factor (VIF) was calculated. Only factor with VIF less than 5 was considered for included in the further multivariate analysis. Hazard ratios (HRs) along with their 95% confidence intervals (CIs) were also calculated. Receiver operating characteristic (ROC) curves were adopted to determine the diagnostic efficacy of CMR parameters to predict primary outcomes. The optimal cut-off values were sought by calculating the Youden index. All participants were divided into higher and lower risk groups according to the optimal cut-off values. A comparison between the higher and lower risk groups was assessed using the log-rank test. The event-free survival probability was plotted using the Kaplan–Meier curve. All statistical analyses were performed on SPSS software (version 26.0, International Business Machines, Armonk, New York, USA). For all calculations, a statistically significant difference was defined as *P* < 0.05 (two sides).

## Results

### Baseline characteristics

**Figure 2:**
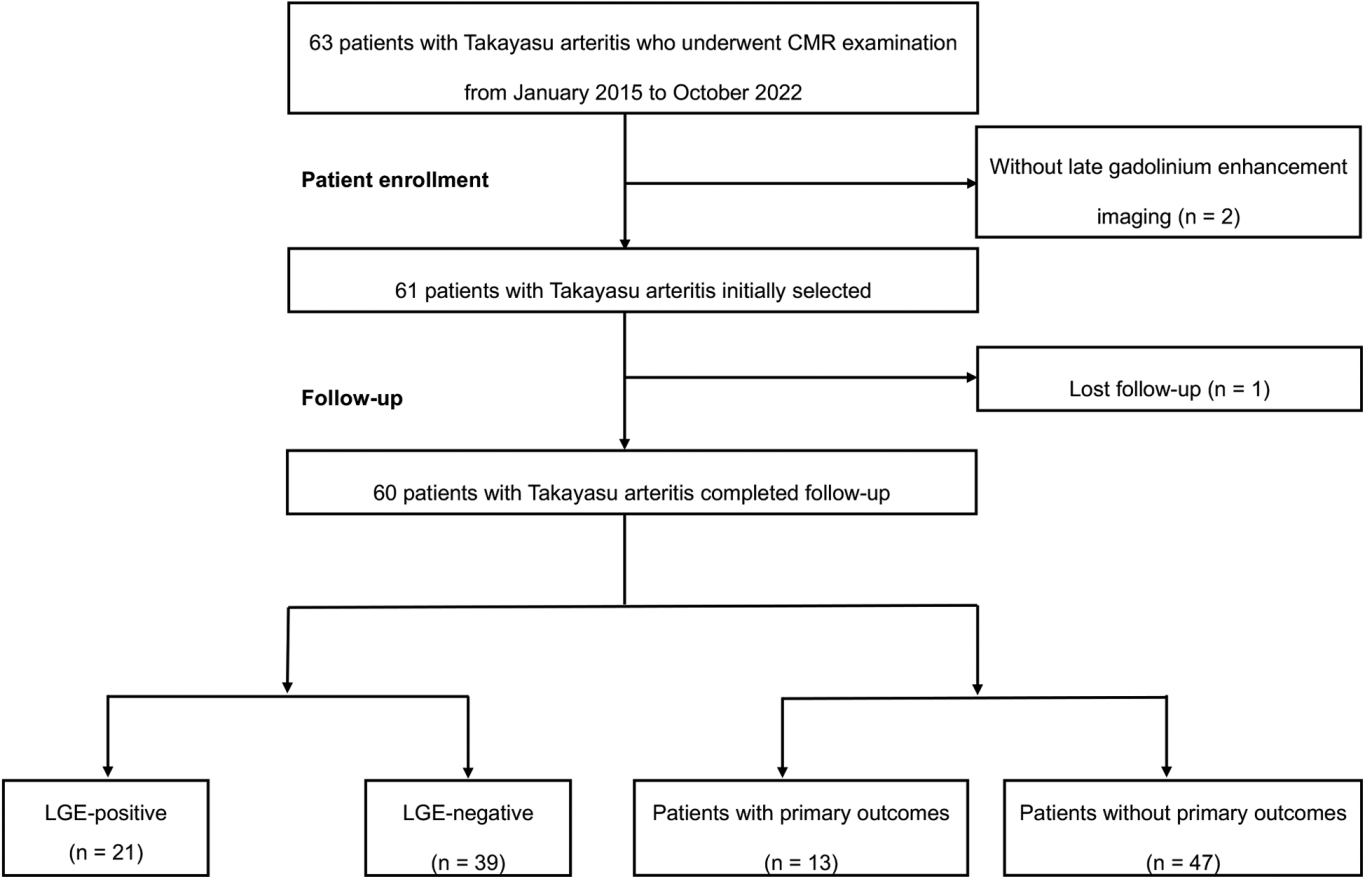
The flowchart of this study. CMR, cardiac magnetic resonance; LGE, late gadolinium enhancement.

The study flowchart of patient enrollment is presented in Figure 2. A total of 63 consecutive patients with TA undergoing a CMR examination between January 2015 and October 2022 were retrospectively enrolled. Two patients with non-contrast CMR due to renal dysfunction and one patient lost to follow-up were excluded. Eventually, sixty patients with TA were included in the study cohort.

LGE-positive was found in twenty-one patients (21/60, 35%) with TA, while the remaining were LGE-negative (39/60, 65%). Clinical baseline characteristics of the overall study group, LGE-negative group, and LGE-positive group were summarized in Table 1. The age of total TA patients was 38.2 ± 13.8 years and was predominantly female (54/60, 90.0%). Patients in the LGE-positive group were older compared with patients in the LGE-negative group (44.5 ± 16.3 vs. 34.9 ± 11.1 years; *P* = 0.027). No statistically significant differences were detected between the LGE-negative and LGE-positive groups regarding demographics and clinical parameters (all *P* > 0.05).

**Table 1:** Baseline characteristics of the overall study group, LGE-negative group, and LGE-positive group.

	Overall (*n* = 60)	LGE-negative (*n* = 39)	LGE-positive (*n* = 21)	*P* value
Demographics				
Age (years)	38.2 ± 13.8	34.9 ± 11.1	44.5 ± 16.3	0.022
Female, *n* (%)	54 (90.0)	35 (89.7)	19 (90.5)	0.928
BMI (kg/m^2^)	22.0 (20.6–24.9)	21.8 (19.6–23.4)	22.5 (20.8–26.4)	0.052
Clinical parameters				
Heart rate (beats/min)	76.5 (70.3–85.0)	80.0 (72.0–85.0)	75.0 (66.5–83.5)	0.200
Systolic pressure (mmHg)	125.0 (110.3–139.5)	125.0 (110.0–140.0)	122.0 (111.5–140.5)	0.804
Diastolic pressure (mmHg)	70.0 (62.3–80.0)	72.0 (64.0–83.0)	68.0 (60.5–78.0)	0.100
Medical history				
Smoking, *n* (%)	7 (11.7)	4 (10.3)	3 (14.3)	0.647
Alcoholic, *n* (%)	3 (5.0)	3.0 (7.7)	0 (0)	0.105
Hypertension, *n* (%)	32 (53.3)	20 (51.3)	12 (57.1)	0.664
Diabetes, *n* (%)	7 (11.7)	3 (7.7)	4 (19.0)	0.202
Hypertriglyceridemia, *n* (%)	13 (21.7)	8 (20.5)	5 (23.8)	0.769
Arrhythmia, *n* (%)	9 (15.0)	4 (10.3)	5 (23.8)	0.170

Data were expressed as mean ± SD or median (interquartile range) or numbers (percentages).

LGE, late gadolinium enhancement; BMI, body mass index.

### Morphological and functional parameters of CMR

Morphological and functional parameters of CMR in the overall study group, LGE-negative group, and LGE-positive group are displayed in Table 2. No statistically significant difference (*P* = 0.089) was detected in LVWT between the LGE-negative and LGE-positive groups. For LV functional parameters, lower LVEF (*P* = 0.033), higher LV end-diastolic volume index (LVEDVI; *P* = 0.033) and end-systolic volume index (LVESVI; *P* = 0.012) were observed in the LGE-positive group compared with the LGE-negative group. Moreover, increased LV mass index (LVMI; *P* = 0.008) was also noticed in the LGE-positive group compared with the LGE-negative group. However, LV stroke volume index and RV functional parameters did not exhibit statistically significant differences (all *P* > 0.05) between the LGE-negative and LGE-positive groups.

**Table 2:** Morphological and functional parameters and tissue characteristics of CMR in the overall study group, LGE-negative group, and LGE-positive group.

Parameters	Overall (*n* = 60)	LGE-negative (*n* = 39)	LGE-positive (*n* = 21)	*P* value
Morphology				
LVWT (mm)	9.1 (7.7–11.6)	9.1 (7.7–10.7)	9.5 (7.9–12.4)	0.398
Function				
LVEF (%)	59.0 (47.4–64.0)	60.3 (52.6–63.8)	47.3 (38.8–64.5)	0.033
LVEDVI (ml/m^2^)	77.6 (62.0–103.9)	70.9 (61.3–88.0)	104.2 (70.0–116.2)	0.008
LVESVI (ml/m^2^)	30.3 (22.0–49.2)	27.9 (21.6–37.8)	48.8 (25.0–66.6)	0.012
LVSVI (ml/m^2^)	42.6 (36.7–52.9)	42.9 (36.4–52.9)	42.2 (38.5–51.0)	0.944
LVMI (g/m^2^)	50.9 (38.9–64.5)	45.7 (37.7–58.6)	57.1 (44.1–77.8)	0.008
RVEF (%)	54.1 (45.6–60.8)	55.4 (48.6–60.8)	51.6 (37.3–59.9)	0.143
RVEDVI (ml/m^2^)	62.9 (54.7–76.3)	63.0 (55.0–76.4)	62.2 (54.4–75.9)	0.659
RVESVI (ml/m^2^)	29.6 (22.6–37.6)	26.8 (22.3–37.8)	31.8 (24.2–37.4)	0.390
RVSVI (ml/m^2^)	34.3 (26.0–42.5)	34.3 (28.2–43.5)	30.8 (21.6–39.7)	0.087
Tissue characteristics				
LGE segment			6.0 (2.0–9.0)	
LGE location in LV				
Anterior wall	9 (15.0)		9 (42.9)	
Septal wall	18 (30.0)		18 (85.7)	
Inferior wall	9 (15.0)		9 (42.9)	
Lateral wall	12 (20.0)		12 (57.1)	
LGE distribution in LV				
Subendocardial	7 (11.7)		7 (33.3)	
Middle wall	12 (20.0)		12 (57.1)	
Subepicardial	1 (1.7)		1 (4.8)	
Transmural	5 (8.3)		5 (23.8)	
LGE pattern in LV				
Line	5 (8.3)		5 (23.8)	
Patchy	7 (11.7)		7 (33.3)	
Infarction	10 (16.7)		10 (47.6)	

Data were expressed as median (interquartile range) or numbers (percentages).

CMR, cardiac magnetic resonance; LGE, late gadolinium enhancement; LVWT, left ventricular wall thickness; LVEF, left ventricular ejection fraction; LVEDVI, left ventricular end-diastolic volume index; LVESVI, left ventricular end-systolic volume index; LVSVI, left ventricular stroke volume index; LVMI, left ventricular mass index; RVEF, right ventricular ejection fraction; RVEDVI, right ventricular end-diastolic volume index; RVESVI, right ventricular end-systolic volume index; RVSVI, right ventricular stroke volume index.

### Characteristics of LGE

The general characteristics of LGE in the overall study group and LGE-positive group are shown in Table 2. There were 6.0 (IQR: 2.0–9.0) LGE segments per TA patient in the LGE-positive group. For the location of the LGE, the LGE was observed in the anterior wall (9/21, 42.9%), the septal wall (18/21, 85.7%), the inferior wall (9/21, 42.9%), and the lateral wall (12/21, 57.1%), with the septal wall predominantly. For the distribution of LGE, middle wall LGE was observed in twelve of twenty-one patients (12/21, 57.1%), followed by subendocardial LGE (7/21, 33.3%), transmural LGE (5/21, 23.8%), and subepicardial LGE (1/21, 4.8%). For the pattern of LGE, line, patchy, and infarction patterns were observed in five (5/21, 23.8%), seven (7/21, 33.3%), and ten (10/21, 47.6%) of twenty-one patients, respectively.

**Figure 3:**
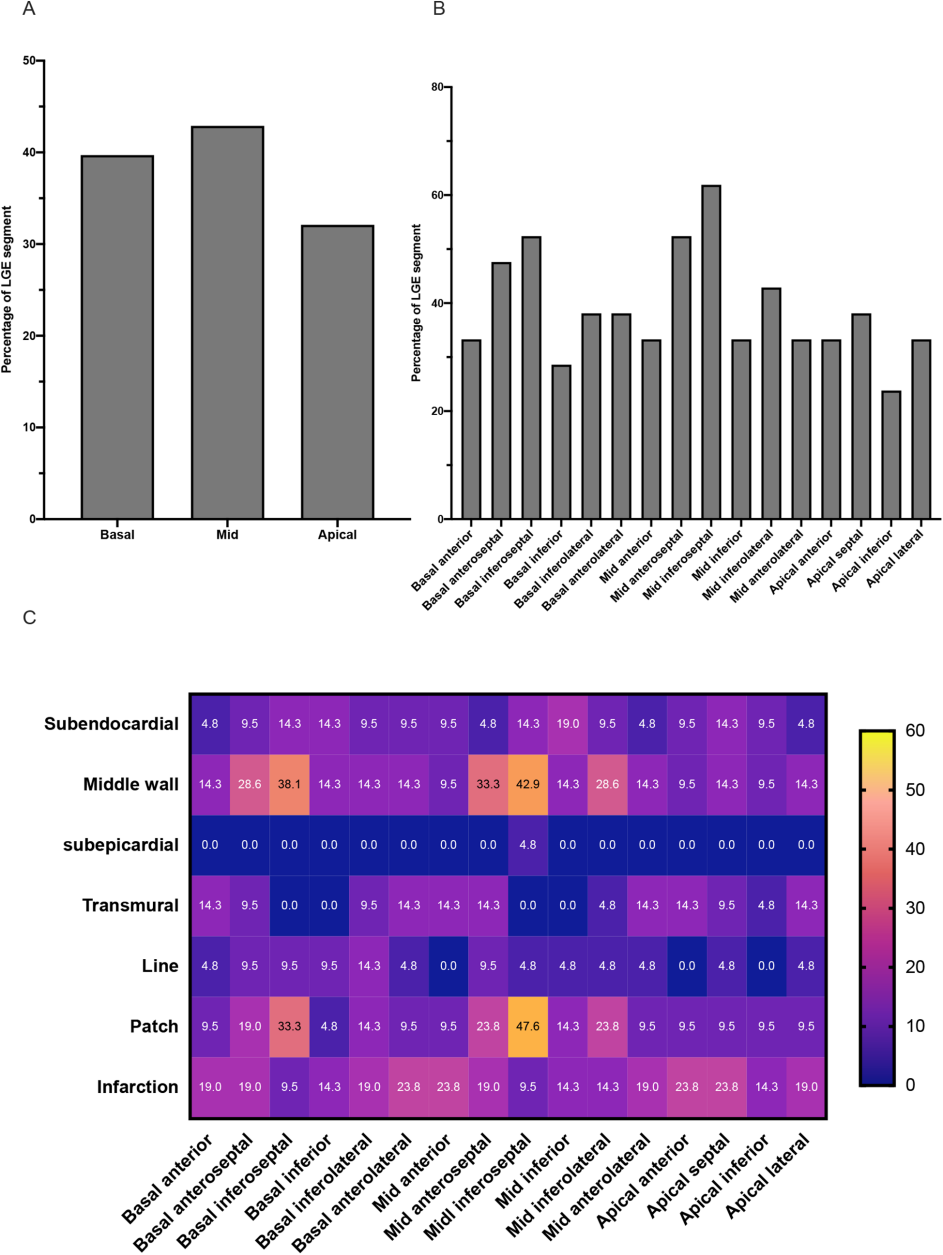
Characteristics of LGE in TA patients of the LGE-positive group. **(A)** The graph shows the incidence of LGE segments at the level of the basal, middle, and apical segments of the left ventricle. **(B)** The graph shows the incidence of LGE in each of the sixteen segments of the left ventricle. **(C)** The heat map shows the distribution and pattern features of LGE in each of the sixteen segments of the left ventricle.

The sixteen segment characteristics of LGE in the LGE-positive group are displayed in Figure 3. For the patients of the LGE-positive group, LGE-positive was detected in 131 (131/336, 39.0%) of the total 336 segments. In terms of distribution, subendocardial LGE and middle-wall LGE are more common. Moreover, the prevalence of patchy LGE and infarction LGE patterns was higher compared with line pattern in the LGE-positive group.

### Follow-up and prognosis

During a median follow-up period of 1,892 days (IQR: 1,764–1,988 days), the primary outcomes were recorded in thirteen patients with TA (13/60, 21.7%), with cardiovascular death occurred in four patients (4/60, 6.7%), hospitalization for heart failure in five patients (5/60, 8.3%), coronary revascularization in four patients due to coronary atherosclerotic disease (4/60, 6.7%), and no patient experienced stroke (0/60, 0%).

Univariate and multivariate Cox proportional hazards regression analyses for primary outcomes in the total patients with TA are displayed in Table 3. Univariate Cox regression analysis showed that the LVWT (HR = 1.180, 95%CI: 1.019–1.388; *P* = 0.028), LVEF (HR = 0.949, 95%CI: 0.920–0.979; *P* = 0.001), LVEDVI (HR = 1.014, 95%CI: 1.001–1.027; *P* = 0.040), LVESVI (HR = 1.016, 95%CI: 1.004–1.029; *P* = 0.003), LVMI (HR = 1.030, 95%CI: 1.013–1.048; *P* = 0.001), and LGE-positive (HR = 4.478, 95%CI: 1.376–14.570; *P* = 0.013) were independently associated with the primary outcomes, respectively.

**Table 3:** Univariate and multivariate cox proportional hazards regression analyses for primary outcomes in patients with TA.

Variates	Univariate analysis			Multivariate analysis		
	HR	95%CI	*P* value	HR	95%CI	*P* value
Age	1.036	0.999–1.075	0.060			
Gender	1.757	0.389–7.942	0.464			
BMI	1.132	0.946–1.356	0.176			
Heart rate	0.958	0.909–1.011	0.117			
Systolic pressure	1.019	0.991–1.048	0.182			
Diastolic pressure	0.995	0.957–1.035	0.816			
Smoking	2.451	0.674–8.912	0.174			
Alcoholic	1.866	0.242–14.370	0.549			
Hypertension	1.350	0.442–4.127	0.599			
Diabetes	1.620	0.359–7.319	0.531			
Hypertriglyceridemia	0.590	0.131–2.666	0.493			
Arrhythmia	1.031	0.228–4.655	0.968			
LVWT (mm)	1.180	1.019–1.388	0.028			
LVEF (%)	0.949	0.920–0.979	0.001			
LVEDVI (ml/m^2^)	1.014	1.001–1.027	0.040			
LVESVI (ml/m^2^)	1.016	1.004–1.029	0.007			
LVSVI (ml/m^2^)	0.976	0.930–1.024	0.319			
LVMI (g/m^2^)	1.030	1.013–1.048	0.001	1.030	1.013–1.048	0.001
LGE-positive	4.478	1.376–14.570	0.013			
RVEF (%)	0.974	0.937–1.012	0.172			
RVEDVI (ml/m^2^)	0.976	0.938–1.015	0.226			
RVESVI (ml/m^2^)	0.999	0.960–1.040	0.960			
RVSVI (ml/m^2^)	0.951	0.902–1.004	0.067			

Multivariate analysis adjust for age, gender, LVWT, LVEF, and LGE-positive.

TA, takayasu arteritis; HR, hazard ratio; CI, confidence interval; BMI, body mass index; LVWT, left ventricular wall thickness; LVEF, left ventricular ejection fraction; LVEDVI, left ventricular end-diastolic volume index; LVESVI, left ventricular end-systolic volume index; LVSVI, left ventricular stroke volume index; LVMI, left ventricular mass index; LGE, late gadolinium enhancement.

**Figure 4:**
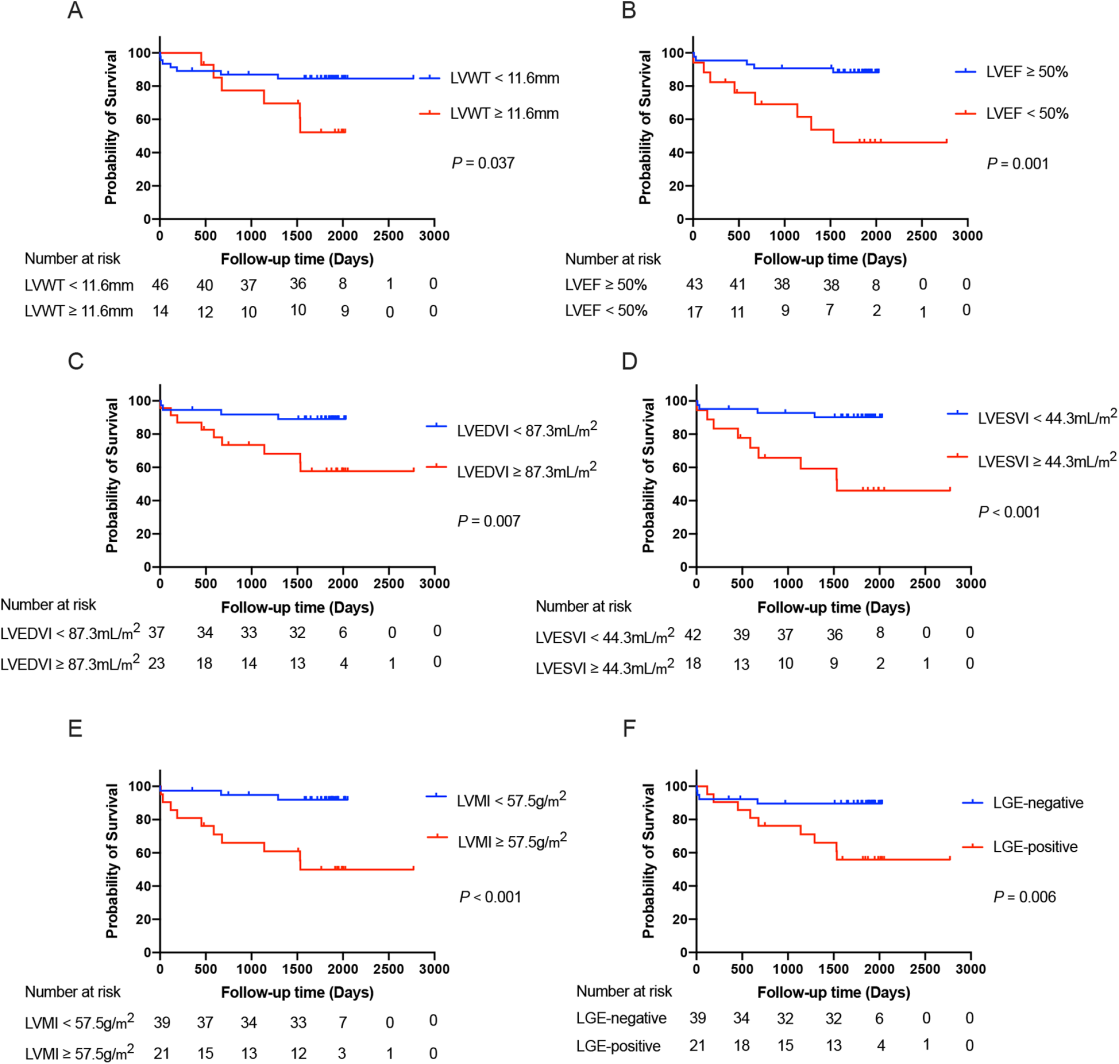
The Kaplan–Meier survival curves grouped according to the cut-off values of LVWT **(A)**, LVEF **(B)**, LVEDVI **(C)**, LVESVI **(D)**, and LVMI **(E)**, as well as the status of the LGE **(F)**, respectively, in the total patients with TA. LVWT, left ventricular wall thickness; LVEDVI, left ventricular end-diastolic volume index; LVESVI, left ventricular end-systolic volume index; LVEF, left ventricular ejection fraction; LVMI, left ventricular mass index; LGE, late gadolinium enhancement.

The determination of cut-off values for the CMR parameters that were statistically significant in the univariates analysis (including only LVWT, LVEDVI, LVESVI, and LVMI) was displayed in Supplementary Table S1. After that, survival curves were plotted based on the cut-off values of LVWT, LVEDVI, LVESVI, and LVMI divided into two subgroups of higher and lower levels. Similarly, subgroups based on LVEF and the status of the LGE were also categorized into LVEF ≥ 50% vs. <50% and those with LGE-negative vs. LGE-positive, respectively. The Kaplan–Meier curves drawn for the above subgroups are shown in Figure 4.

After adjustment for age and gender, we included LVWT, LVEF, LVMI, and LGE-positive as covariates in the multivariate analysis. However, only LVMI (HR = 1.030, 95%CI: 1.013–1.048; *P* = 0.001) maintained an independent predictor of the primary outcome in further multivariate analysis. Based on the optimal predictive value of LVMI, all TA patients were categorized into two subgroups with LVMI < 57.5 g/m^2^ vs. ≥57.5 g/m^2^. The Kaplan–Meier survival plot based on subgroups of LVMI was also depicted in Figure 4. The event-free survival probability was significantly lower for TA patients with LVMI ≥ 57.5 g/m^2^ compared with patients with LVMI < 57.5 g/m^2^ (log-rank *P* < 0.001).

## Discussion

We investigated the prevalence and characteristics of LGE of the LV myocardium and its prognostic value using CMR imaging in patients with TA. This study found that LGE-positive was observed in 35% of total TA patients. LGE is mainly distributed in the middle wall and subendocardial region, showing typical patchy and infarcted pattern features. Patients with LGE-positive have higher LV myocardial mass and worse LV function compared with patients with LGE-negative. In addition, our study further revealed that although LGE-positive is independently associated with the primary outcomes, LGE-positive does not provide independent prognostic information in patients with TA after adjusting for other variables. The LVMI is the strongest independent predictor of the primary outcomes and can help clinicians in risk stratification and prognostic management in patients with TA.

Myocardial fibrosis (MF) is one of the most common histologic features in heart failure due to many cardiovascular diseases. MF is associated with ventricular systolic and diastolic dysfunction, abnormal remodeling, increased ventricular wall stiffness, and adverse cardiac outcomes (19, 20). Because of the higher spatial resolution, CMR has advantages over other imaging modalities in the assessment of myocardial fibrosis (21). LGE imaging with CMR provides information about myocardial tissue characteristic and can detect myocardial fibrosis and scar (22, 23). Different diseases leading to heart failure are associated with different LGE characteristics (24, 25). Our study found that LGE-positive detected by CMR imaging was observed in 35% of total TA patients. In TA patients with LGE-positive, LGE was also found in the septal, lateral, anterior, and inferior wall. This suggests that myocardial damage and abnormalities in myocardial tissue characteristics are present in patients with TA.

In TA patients with LGE-positive, the distribution and pattern of LGE are diverse and different. LGE was predominantly distributed in the middle wall and subendocardial region. In addition, the transmural LGE and subepicardial LGE were also noticed. The incidence of infarction pattern was the highest, and patchy and line LGE were also prevalent. The diverse LGE distributions and patterns reflect the fact that myocardial injury and adverse ventricular remodeling detected by CMR imaging in patients with TA have multiple manifestations.

Our findings revealed that increased LV mass, evaluated LVEDVI and LVESVI, and decreased LVEF in patients with LGE-positive compared with those with LGE-negative. This indicated that adverse LV myocardial remodeling and dysfunction are associated with LGE in patients with TA. This also suggests that it is essential for clinicians to focus on LV myocardial and ventricular remodeling in patients with TA.

### Prognostic value of CMR parameters

Up to now, numerous studies have emerged regarding the value of LGE in the prognostic assessment of cardiovascular disease. Although there are discrepancies between the results of different studies, most have shown that LGE is an independent predictor of adverse cardiovascular events. This has been confirmed in meta-analyses in the populations of suspected or known coronary artery disease (26), hypertrophic cardiomyopathy (27, 28), dilated cardiomyopathy (29–31), systemic amyloidosis (32), cardiac sarcoidosis (33, 34), aortic stenosis (35), and myocarditis (36). In this study, our findings showed that LGE was independently correlated with the primary outcomes in the univariate analysis. However, LGE did not maintain its value as an independent predictor of primary outcomes after various variates were added to the multivariate analysis.

In contrast, LVMI showed important value in providing independent prognostic information in patients with TA in the multivariate analysis. In terms of the predictive value of LGE, our findings are inconsistent with those of previous studies. Actually, this was a surprising and unexpected discovery for us. There are several possible reasons for this inconsistency. First, the sample size and adverse cardiovascular events of our study are relatively small. Therefore, LGE may not be statistically significant in multivariate analysis. Second, TA as a systemic and complex disease, cardiac abnormalities due to TA can manifest in different disease forms. The visual observation of LGE may be influenced by a variety of diseases. We speculate that LGE may reflect adverse and late myocardial remodeling, whereas LVMI may reflect compensatory and early myocardial remodeling. For the prognostic assessment of cardiovascular abnormalities due to TA, LVMI may be a more sensitive and stronger independent predictor of prognosis compared with LGE in patients with TA. However, this needs to be confirmed by further large-sample study cohorts and echocardiography studies.

### Limitations

We realize some limitations of this study. First, TA is a rare systemic disease and predominantly female, but this study only retrospectively included a relatively small number of patients with TA from one hospital, which may overestimate the occurrence of LGE-positive due to selection bias. Also, because of the limitations of sample size and number of outcomes, this study did not include too many factors to avoid the problem of overfitting in the univariate and multivariate Cox proportional hazards regression analyses. The predictive value of LGE needs to be further confirmed by prospective studies with multicenter and large samples. Second, although a blinded interpretation of LGE was applied, the difference in LGE image quality due to different scanner equipment and parameters may still exist. Third, LGE imaging has limitations in detecting diffuse myocardial fibrosis. Indeed, patients with LGE-negative may have mild diffuse myocardial fibrosis due to increased LV afterload. However, this requires further T1 mapping imaging and analysis. Unfortunately, most of the patients in this study did not undergo a T1 mapping scan. Therefore, T1 mapping remains promising for detecting early myocardial remodeling and prognostic assessment but awaits future investigation.

## Conclusions

LGE-positive on CMR imaging was observed in 35% of patients with TA. LGE was associated with worse LV function and adverse LV remodeling. It is the LVMI rather than the LGE that provides independent prognostic information and contributes to risk stratification and prognostic management in patients with TA.

## Data Availability

The raw data supporting the conclusions of this article will be made available by the authors, without undue reservation.
