# Supplementary material for: Alteration of cardiac structure and function and its prognostic value in patients with Takayasu arteritis: a cardiac magnetic resonance study
Source: Front Cardiovasc Med. 2024 Sep 19;11:1475535. doi: 10.3389/fcvm.2024.1475535 (PMC11446742; doi:10.3389/fcvm.2024.1475535)
Supplement: Supplementary file 1 [file Table1.docx]

**Table S1 ROC analysis for LVWT, LVEDVI, LVESVI, and LVMI predicting primary outcomes in total TA patients.**

| **Parameters** | **Cut-off value** | **Sensitivity** | **Specificity** | **AUC** | **95%CI** | ***P* value** |
| --- | --- | --- | --- | --- | --- | --- |
| LVWT (mm) | 11.6 | 53.8% | 83.0% | 0.696 | 0.529–0.863 | 0.032 |
| LVEDVI (mL/m^2^) | 87.3 | 69.2% | 70.2% | 0.660 | 0.472–0.847 | 0.008 |
| LVESVI (mL/m^2^) | 44.3 | 69.2% | 80.9% | 0.732 | 0.581–0.882 | 0.011 |
| LVMI (g/m^2^) | 57.5 | 76.9% | 76.6% | 0.763 | 0.597–0.928 | 0.004 |

Abbreviations: ROC, receiver operating characteristic; LVEDVI, left ventricular end-diastolic volume index; LVESVI, ﻿left ventricular end-systolic volume index; LVMI, left ventricular mass index; TA, Takayasu arteritis; AUC, area under the curve; CI, confidence interval.
